# Animal models of post-traumatic stress disorder: face validity

**DOI:** 10.3389/fnins.2013.00089

**Published:** 2013-05-31

**Authors:** Sonal Goswami, Olga Rodríguez-Sierra, Michele Cascardi, Denis Paré

**Affiliations:** ^1^Center for Molecular and Behavioral Neuroscience, Rutgers State UniversityNewark, NJ, USA; ^2^Child Advocacy and Policy, Center for Child Advocacy, Montclair State UniversityMontclair, NJ, USA

**Keywords:** post-traumatic stress disorder, animal model, recognition memory, extinction, predator threat

## Abstract

Post-traumatic stress disorder (PTSD) is a debilitating condition that develops in a proportion of individuals following a traumatic event. Despite recent advances, ethical limitations associated with human research impede progress in understanding PTSD. Fortunately, much effort has focused on developing animal models to help study the pathophysiology of PTSD. Here, we provide an overview of animal PTSD models where a variety of stressors (physical, psychosocial, or psychogenic) are used to examine the long-term effects of severe trauma. We emphasize models involving predator threat because they reproduce human individual differences in susceptibility to, and in the long-term consequences of, psychological trauma.

## Post-traumatic stress disorder

### Clinical diagnosis, precipitating event, and heritability studies

Since the usefulness of animal models of **post-traumatic stress disorder** (PTSD) depends on their ability to reproduce the human syndrome, we first summarize the diagnostic features of this anxiety disorder based on the criteria provided in the *Diagnostic and Statistical Manual for Mental Disorders IV-TR* (American Psychiatric Association, 2000). PTSD is an anxiety disorder triggered by exposure to a traumatic event. Depending on the type of trauma and its intensity, the proportion of individuals who develop PTSD varies greatly. For instance, traumatic events of human design (i.e., violent crime, rape, war) lead to a higher incidence of PTSD than natural disasters (North et al., 2012).

To receive a diagnosis of PTSD, the individual must experience a traumatic event that produces feelings of intense fear, horror, or helplessness. Once this criterion is met, individuals must pass a symptom threshold for each of three symptom clusters: re-experiencing, avoidance, and hyperarousal. (1) Re-experiencing occurs when individuals involuntary re-live the traumatic event in a variety of ways, including flashbacks and recurrent nightmares. These sleep disturbances may explain some of the cognitive impairments reported in PTSD (van Liempt et al., 2011, 2013). (2) The avoidance symptom cluster includes individuals' efforts to avoid and emotionally detach themselves from people, places, and situations that remind them of the traumatic event. (3) Hyperarousal is characterized by heightened physiological reactivity as evidenced by exaggerated startle response, difficulty concentrating, and hypervigilance. Symptoms must occur for at least 1 month and cause significant impairment in the individual's functioning.

Not every individual who experiences a traumatic event suffers from PTSD. Individual variations are partly explained by a genetic contribution to this anxiety disorder. For instance, the correlation of PTSD status is higher among monozygotic than dizygotic twins (Nugent et al., 2008; Afifi et al., 2010). Consistent with this, genetic studies identified DNA variations that show a strong association with PTSD status and likely confer susceptibility/resilience to some individuals (reviewed in Mahan and Ressler, 2012). Interestingly, PTSD heritability coincides with that of other psychiatric conditions such as generalized anxiety and depression (Chantarujikapong et al., 2001; Fu et al., 2007), suggesting that these disorders gain expression through common biological mechanisms. Indeed, in the National Comorbidity Study, roughly one-half of men and women who suffered PTSD also met diagnostic criteria for major depressive disorder (Brady et al., 2000).

### Abnormal fear regulation in PTSD

In PTSD, the fear responses triggered by the initial trauma are repetitively re-experienced, often through flashbacks and recurring nightmares. Moreover, fear tends to generalize to other stimuli and situations, contributing to avoidance of fear-provoking places, activities, and people. Consistent with this, numerous laboratory studies support the view that fear is regulated abnormally in PTSD (for instance see: Orr et al., 2000; Peri et al., 2000; Guthrie and Bryant, 2006; Milad et al., 2008; Glover et al., 2011). Below, we briefly review this evidence.

The leading experimental model to study how organisms learn to predict danger based on experience is **classical fear conditioning** (Ledoux, 2000). In this model, a neutral conditioned stimulus (CS), such as a context or tone, is paired with a noxious unconditioned stimulus (US), typically a mild electrical shock to the hand or wrist. After a few CS-US pairings, presentation of the CS alone comes to elicit conditioned fear responses (e.g., galvanic skin conductance, pupil dilation). As discussed below, a similar network of brain structures regulate fear learning in humans and animals (Phelps and Ledoux, 2005).

While it is clear that fear is abnormally regulated in PTSD, the evidence is mixed as to whether individuals with PTSD acquire and/or express stronger conditioned fear responses than controls (Morgan et al., 1995; Grillon et al., 1998; Orr et al., 2000; Kumari et al., 2001; Blechert et al., 2007; Norrholm et al., 2011; Jovanovic et al., 2012). In contrast, there is consensus that those with PTSD display increased baseline startle responses (Morgan et al., 1995; Grillon et al., 1998; Kumari et al., 2001). It has been proposed that this form of fear dysregulation results from an inability of individuals with PTSD to differentiate safe from threatening contexts (Grillon et al., 1998). Indeed, (Grillon, 2002) found that when individuals with PTSD are confronted with situations perceived as stressful, and presented with unpredictable adverse events, they exhibit potentiated startle responses compared to controls. These findings support the role of unrealistic danger expectations, which can contribute to a chronic state of anxiety that allows fear to generalize to previously safe situations and progressively invade more aspects of an individual's life.

Also, subjects who suffer from PTSD are deficient at learning that stimuli previously associated with adverse outcomes no longer present a threat (Orr et al., 2000; Peri et al., 2000; Rothbaum et al., 2001; Guthrie and Bryant, 2006; Milad et al., 2008; Norrholm et al., 2011; Jovanovic et al., 2012). As a result, PTSD is often characterized as a failure to learn safety in the absence of threat (Milad et al., 2006). In the laboratory, this form of learning is modeled by repeatedly presenting the CS in the absence of the US. This process, termed **extinction**, is closely related to an approach commonly used by clinicians to treat PTSD: prolonged exposure therapy (PE; Bisson et al., 2007; Powers et al., 2010; Rauch et al., 2012). In PE, individuals with PTSD are presented with actual or imagined trauma reminders depicting the most feared aspects of the traumatic event. These cues, analogous to the CS, are not followed by danger (or US). With sufficient repetition and when paced appropriately, extinction occurs and PTSD symptoms remit. Note that extinction training does not reverse or erase the original fear memory, but leads to the formation of a new inhibitory memory (CS-no US) that competes with the original CS-US association for control of behavior (Myers and Davis, 2002).

While extinction deficits can be remedied with PE, do the extinction deficits characteristic of PTSD predate or result from trauma? A study of monozygotic twins discordant for trauma exposure addressed this question. Milad et al. (2008) found that PTSD is associated with impaired extinction retrieval *after* but not before trauma. This suggests that the extinction deficit is not a pre-existing condition; rather it develops as a result of trauma (Milad et al., 2008).

Consistent with the abnormal fear regulation seen in PTSD, two brain structures critically involved in the acquisition and extinction of conditioned fear responses, the amygdala and ventromedial pre-frontal cortex (vmPFC), show atypical activity patterns in PTSD. Indeed, results from several functional imaging studies show that reminders of traumatic events activate the amygdala more strongly in individuals with PTSD than in controls (Shin et al., 2006). Additional evidence of the amygdala's involvement in PTSD comes from a retrospective study of Vietnam war veterans who suffered localized brain injuries: participants with amygdala damage had a dramatically lower incidence of PTSD than subjects with lesions to other parts of their brain (Koenigs et al., 2008). Further strengthening the link between aberrant amygdala activation and PTSD, in a prospective study, higher activation of the amygdala in response to emotional images before trauma predicted reports of higher post-traumatic stress symptom severity (Admon et al., 2009). In contrast, in individuals with PTSD, vmPFC was hypo-responsive and smaller in volume than in controls (Bremner et al., 2008). Given the critical role played by vmPFC in extinction, these observations suggest that hypo-activity in this region may be responsible for the extinction deficits exhibited by humans with PTSD (Milad et al., 2008).

### Abnormal hippocampal structure and function

The hippocampus plays a critical role in the formation of declarative memories and is rich in receptors for glucocorticoids, a class of steroid hormones released by the adrenal glands during stressful conditions. High levels of circulating stress hormones lead to cellular atrophy in the hippocampus (Sapolsky, 2000; McEwen, 2007), inhibition of hippocampal neurogenesis (Gould et al., 1997) and of activity-dependent synaptic plasticity (Pavlides et al., 2002; Popoli et al., 2011). Moreover, stress impairs hippocampal-dependent memory retrieval in humans (De Quervain et al., 2000; Wolf et al., 2001), an effect mediated in part by direct glucocorticoid effects in the hippocampus (Roozendaal et al., 2003).

Consistent with this, individuals with PTSD have smaller hippocampal volumes (Gilbertson et al., 2002; Bremner et al., 2003; Kitayama et al., 2005; Wang et al., 2010) and exhibit impaired performance on hippocampal-dependent tasks (for instance, see Shin et al., 2004; Lindauer et al., 2006; Gilbertson et al., 2007; Thomaes et al., 2009; Hayes et al., 2011; reviewed in Samuelson, 2011). Interestingly, studies of monozygotic twins discordant for combat exposure indicate that the abnormalities in hippocampal structure and function seen in individuals with PTSD are present in their non-traumatized co-twins, suggesting that they predate onset of the disorder (Gilbertson et al., 2002, 2007). In particular, Gilbertson et al. (2007) examined the impact of PTSD on performance in **allocentric** spatial tasks that rely on the identification of spatial relationships between neighboring stimuli. These tasks cannot be solved using an **egocentric** frame of reference and are believed to depend on hippocampal functioning (Langston and Wood, 2010). In monozygotic twins discordant for combat exposure, individuals with PTSD and their non-traumatized co-twin were impaired on allocentric tasks relative to non-PTSD control twins (Gilbertson et al., 2007). This contrasts with the **fear extinction** deficit reviewed above, which develops *after* trauma.

The significance of these hippocampal deficits for the etiology of PTSD comes from earlier studies of learning tasks that engage hippocampal-dependent, “cognitive” vs. striatal-dependent, “habit” memory systems (reviewed in Poldrack and Packard, 2003). These systems interact in a competitive manner such that lesions or inactivation of one system leads the subject to favor the other, an interpretation supported by functional imaging studies in humans (Poldrack et al., 1999). Particularly relevant to PTSD is the observation that in such dual-solution memory tasks, stress biases rats (Kim et al., 2001; Packard and Wingard, 2004) and humans (Schwabe et al., 2007, 2008) toward striatal-dependent learning strategies. Moreover, the basolateral amygdala mediates these effects of stress (Wingard and Packard, 2008). Therefore, it is possible that the antecedent hippocampal deficits and amygdala hyper-responsiveness seen in PTSD lead to a greater engagement of the striatum and automatic responding to trauma-related cues (Goodman et al., 2012). In addition, since the hippocampus is required for differentiating contexts (Rudy et al., 2004), its impaired functioning might interfere with the contextual regulation of fear responses, promoting fear generalization.

## Animal models of PTSD

Animal models provide an important avenue for studying the pathophysiology of PTSD because they circumvent ethical limitations associated with human research in three important ways. First, in contrast with human studies where participants cannot be randomly assigned to trauma exposure, in animal models researchers can manipulate all aspects of the stressor including type, timing, and intensity. Second, these manipulations allow investigators to separate pre-existing factors from those that are acquired after exposure to the stressor. Finally, animal models permit the use of more invasive techniques than ethically acceptable in humans.

Generally, the validity of animal models is assessed using a combination of criteria including: (1) Face validity—does the model reproduce symptoms associated with the human syndrome? (2) Construct validity—does the model measure what it intends to measure (e.g., EPM as a measure of anxiety)? (3) Predictive validity—can the model predict treatment (e.g., drugs) outcomes seen in the human syndrome? (4) Discriminant validity—does the model differentiate between those with and without PTSD? (Siegmund and Wotjak, 2006; Belzung and Lemoine, 2011). This review will focus on assessing the face validity of animal models of PTSD.

Fortunately, much work has focused on defining animal models of PTSD that reproduce salient features of the human syndrome (see Adamec et al., 2006; Cohen et al., 2006a; Siegmund and Wotjak, 2006). In these models, animals are exposed to various types of stressors, leading to long-lasting changes in circulating levels of stress hormones and/or in anxiety-like behaviors, assessed with standardized tests such as the elevated plus maze (EPM), open field, social interaction test, and acoustic startle. Stressors can vary along many dimensions including duration (acute vs. chronic), controllability (controllable vs. uncontrollable), and frequency (single vs. repeated). Below, we describe the most common rodent PTSD models, which we organized based on the type of stressor. In section “Ecological Validity of Rodent PTSD Models,” we consider whether the various models reproduce salient features of human PTSD. Attention is focused on individual differences in trauma susceptibility, deficits in fear extinction, and impaired hippocampal functioning. A thorough review of this literature exceeds the format of this article; therefore, we provide an overview of key findings.

### Models using physical stressors

The traumatic events that precipitate PTSD in humans often involve potentially life-threatening bodily harm. Similarly, many animal models of PTSD use **physical stressors** such as inescapable footshocks, underwater/forced swim paradigms, immobilization/restraint stress, or a combination of multiple stressors.

Delivery of inescapable and unsignaled *footshocks* leads to the formation of robust fear associations to the context where the shocks were administered (Rudy et al., 2004). In addition, some studies report that delivery of numerous (2–10), high intensity (~1.5 mA) footshocks augments anxiety-like behavior on the EPM (Armario et al., 2008) and increases circulating levels of ACTH and corticosterone (CORT; Daviu et al., 2012). It is important to note that footshock stress is also used to model depression (Seligman and Maier, 1967). Consistent with this, administration of antidepressants attenuates the long-term behavioral effects of inescapable footshocks (e.g., enhanced acoustic startle) (Manion et al., 2007). This finding highlights a major confound associated with many animal models of PTSD relying on physical stressors: it is unclear whether they model PTSD and/or depression. However, PTSD and depression often coexist in humans. Therefore, this is not necessarily a weakness.

*Underwater trauma* has also been used in rats to model the trauma that precipitates PTSD in humans. Here, rats subjected to underwater trauma (40 s swim and 20 s submersion) show an immediate and persistent increase in anxiety in the EPM compared to rats that swim without submersion (Moore et al., 2012).

*Restraint stress*, which involves placing rats in restraining tubes for 2–6 h, leads to increased manifestations of anxiety in the EPM (Vyas et al., 2002), and changes in neuronal morphology within brain regions mediating fear and anxiety (Miller and McEwen, 2006). A related procedure is immobilization stress, where rodents are restrained onto a wooden platform either acutely (single session) or chronically (several sessions). Immobilization stress produces a long-term desensitization of the hypothalamic-pituitary-adrenal (HPA) axis activity to subsequent exposure to the same immobilization stressor (homotypic stressor), but sensitized HPA responsiveness (increased plasma ACTH and CORT levels) to novel (heterotypic) stressors (Armario et al., 2004, 2008; Belda et al., 2008, 2012). However, this is not always the case using other stressors (see below).

*Single prolonged stress* (SPS), contrary to what this name implies, actually involves the administration of *various* stressors (restraint stress, forced swim, and ether exposure). Although SPS does not address the unique contributions of each stressor independently or potential confounds associated with repetitive stress, it is used widely and features some similarities with human PTSD (discussed below; Liberzon et al., 1997; Yamamoto et al., 2009; Takei et al., 2011; Knox et al., 2012; Pitman et al., 2012).

An even more intense approach is the “*variable stress*” models. For example, chronic variable stress (CVS) involves exposing animals to a different stressor daily for 6 days, and this procedure is repeated for several weeks in a row (Molina et al., 1990; McGuire et al., 2010). CVS leads to decreased time on the open arms of the EPM (Zurita et al., 2000). Admittedly, this class of models is extreme, yet it probably comes closest to simulate the chronic stress conditions experienced by military personnel in front-line positions.

### Models using psychosocial stressors

In another approach, animals are submitted to a variety of **psychosocial stressors** such as housing instability, social defeat, and social isolation. Recent work with victims of partner abuse supports the ecological validity of housing instability as an important risk factor for humans and animals alike. Indeed, Rollins et al. (2012) found that difficulties maintaining a stable living environment predicted PTSD, even after level of danger was statistically controlled. Researchers reproduce social instability by changing home cages and cage mates daily (Park et al., 2001; Zoladz et al., 2008, 2012). When social instability is chronic, or combined with other stressors, animals exhibit long-lasting anxiety-like behaviors reminiscent of human PTSD symptoms (Zoladz et al., 2008, 2012; Saavedra-Rodriguez and Feig, 2013).

Although social defeat is typically considered a model of depression (Krishnan et al., 2008), it also induces long-term signs of anxiety (Huhman et al., 1992; Huhman, 2006). Many studies relying on this stressor use Syrian hamsters, because unlike rats, hamsters are solitary animals and readily exhibit signs of territorial aggression (Huhman, 2006). Here, an “intruder” animal is placed in the territory of a larger “resident” animal, prompting the resident to attack the intruder. In a typical experiment, resident-intruder pairings occur for 15 min a day over 4 days. On day 5, physical interactions do not occur (a mesh barrier separates the two animals), and stress hormone levels are tested. Plasma levels of ACTH and CORT are elevated among submissive animals, but not the dominant animals (Huhman et al., 1992), suggesting that the hormonal changes do not reflect physical contact, but a more psychological process akin to human victimization by interpersonal violence. Even a single exposure to social defeat can lead to conditioned defeat, whereby previously defeated animals engage in submissive behaviors, even after becoming residents themselves (Jasnow and Huhman, 2001). Conditioned defeat persists a month after the initial social defeat event (Huhman, 2006). Finally, when social defeat was investigated in rodents, it was shown to cause a persistent enhancement of the acoustic startle response (Pulliam et al., 2010), and anxiety-like behavior on the EPM (Narayanan et al., 2011).

### Models using early life stressors

In humans, prior exposure to trauma, particularly during development can cause long-term hormonal abnormalities and increased risk of developing PTSD (Delahanty and Nugent, 2006). Pioneering studies beginning with Harlow's monkeys (Young et al., 1973) produced a large body of research examining the impact of maternal separation and **early life stressors**. Collectively, these studies show that neonatal isolation enhances stress and anxiety responses upon exposure to severe stress later in life (Diehl et al., 2012). When combined with other stressors, social isolation leads to a marked enhancement of anxiety-like behaviors (Imanaka et al., 2006). Moreover, social isolation pre- or post-weaning increases plasma levels of CORT and hypothalamic levels of corticotropin-releasing hormone mRNA (Zhang et al., 2011). Also, juvenile rats exposed to a severe psychological stressor are more likely to develop extreme anxiety-like phenotypes when exposed to the same stressor again in adulthood (Cohen et al., 2006a,b, 2007). This contrasts with studies of immobilization, where repeated exposure to the same stressor causes a desensitization of the stress response (Armario et al., 2004, 2008; Belda et al., 2008, 2012). Nevertheless, in humans (Yehuda et al., 1998a,b) and animals (De Kloet et al., 2005; Cohen et al., 2007; Bazak et al., 2009), exposure to high levels of stress or glucocorticoids early in life pre-disposes individuals to be more susceptible to subsequent stressors. In animals, this susceptibility can be transmitted through generations (Seckl and Meaney, 2006; Vialou et al., 2013).

### Genetic models of PTSD-like symptoms

Interesting genetic models have been developed in rodents including rat or mouse strains that exhibit high trait anxiety and/or marked fear extinction deficits (Landgraf and Wigger, 2002; Camp et al., 2009; Neumann et al., 2011; Holmes and Singewald, 2013). Another noteworthy line of investigation focuses on variations in anxious temperaments in monkeys (Kalin, 2003; Fox et al., 2008). However, the PTSD-like abnormalities associated with these models do not require exposure to trauma and remain outside the scope of this review.

### Models using psychogenic stressors

Although many of the physical stressor models reviewed above have a “psychogenic” component, they primarily involve physical pain or discomfort. In contrast, the ones discussed below involve threat, but usually no pain. In these models, rodents are exposed to species-relevant predators (**predator stress**) or their odor (**predator threat**; Blanchard and Blanchard, 1988; Dielenberg and McGregor, 2001), leading to the development of long-lasting (3 weeks or more) manifestations of anxiety as assessed with the EPM, social interaction test, and acoustic startle (Adamec and Shallow, 1993; Adamec et al., 1998, 2006; Mesches et al., 1999; Blanchard et al., 2003; Hebb et al., 2003; Roseboom et al., 2007; Nanda et al., 2008; Zoladz et al., 2008, 2012).

A major strength of these models is their ecological relevance. Indeed, field studies of animals in their natural habitat have shown that exposure to predators or associated cues lead to increased glucocorticoid levels, and decreased number of offspring (Clinchy et al., 2010). In addition, the rise in maternal glucocorticoid levels appeared to be transmitted through generations (Sheriff et al., 2010). Therefore, predator stress and threat models arguably constitute a better replica of the kind of life-and-death circumstances that precipitate PTSD in humans than physical stressors.

In the predator stress model, rats are exposed to a cat for one or two sessions of up to 45 min each. Typically, the rats are placed in a small protective enclosure that prevents direct physical interactions (e.g., Diamond et al., 1999). However, in some laboratories, no such measures are taken (e.g., Adamec et al., 1998). While live predators constitute a more intense stressor than their odor, the latter is a convenient alternative that allows for greater repeatability and control over trauma intensity. In the predator threat model, rats are presented with predator odors from natural or synthetic sources. Natural odors are usually obtained from felines (cat fur, bedding, litter). The most common synthetic odor is trimethylthiazoline (TMT), a component of fox feces that rats find aversive. While feline odors can be used as US to support contextual fear conditioning (Blanchard et al., 2001), TMT cannot (Wallace and Rosen, 2000). Consistent with this, cat odors and TMT elicit different patterns of responses with cat odors triggering anxiety-like behaviors whereas TMT evokes avoidance responses (Dielenberg and McGregor, 2001).

## Ecological validity of rodent PTSD models

### Importance of individual differences in susceptibility to trauma

A limitation of some animal models of PTSD is that the stressful event affects all animals similarly or individual differences are not reported; all comparisons are between naive vs. trauma-exposed subjects (for notable exceptions see Siegmund et al., 2009 and Krishnan et al., 2007). This is in contrast with human PTSD where only a proportion of trauma-exposed individuals develop the disorder. Animal models that can capture individual differences in trauma vulnerability have greater ecological and face validity because they allow researchers to investigate factors conferring resilience and susceptibility to trauma.

The predator threat model of PTSD reproduces the individual variations in trauma vulnerability seen in humans. Indeed, predator threat exposure leads to the development of extreme manifestations of anxiety (EBMAs) in a proportion of subjects (Cohen et al., 2006a,b). Interestingly, the incidence of EBMAs following predator threat is much higher in the inbred Lewis rat strain (50%) than in Sprague–Dawley rats (20%), and Fisher rats (10%; Cohen et al., 2006a,b). This finding, which was replicated in a different laboratory (Goswami et al., 2010), strongly implicates a genetic contribution toward the development of extreme anxiety-like states, facilitating investigations of gene-environment interactions.

Consistent with the high incidence of susceptible Lewis rats and data implicating abnormal regulation of the HPA axis in human PTSD, Lewis rats exhibit dampened diurnal variations in CORT levels and blunted ACTH and CORT responses to stressors (Sternberg et al., 1992; Dhabhar et al., 1993). Further paralleling human PTSD where the incidence of inflammatory and autoimmune diseases is high (Boscarino, 2004), Lewis rats show an increased propensity to such disorders and are commonly used in experimental analyses of autoimmune diseases. In both human PTSD and Lewis rats, it was proposed that the impaired responsiveness of the HPA axis coupled to excessive activation of the sympathetic nervous system may play a key role in these pathophysiological processes (Boscarino, 2004; Zoladz and Diamond, 2013). In keeping with this, it was reported that administration of glucocorticoids prior to predatory threat reduces the incidence extreme anxiety-like behavior on the EPM (Cohen et al., 2006b), paralleling human studies (Schelling et al., 2006). Similarly, administration of Neuropeptide Y after predator threat causes a long-lasting reduction in the incidence of the extreme anxiety-like phenotype (Cohen et al., 2012).

The high incidence of the PTSD-like phenotype (~50%) in Lewis rats is advantageous in many ways. Because random groups of Lewis rats include a nearly equal fraction of susceptible and resilient subjects, fewer rats have to be studied to compare the two groups on any dimension. This is particularly advantageous for labor-intensive studies. Along the same lines, because predator threat induces a bimodal distribution of anxiety-like behaviors, there is no need to eliminate animals from the analyses in order to examine only extreme responders as is sometimes done in other stress models. Furthermore, tests can be conducted before or after exposure of the rats to predators or their odor to determine if differences between susceptible and resilient animals predate or are a consequence of the stressor. In one approach for instance, 1 week following a single 10-min exposure to soiled cat litter, Lewis rats are tested on the EPM (Cohen et al., 2006a,b; Goswami et al., 2010, 2012). Rats with extremely compromised exploratory behavior (0 time spent on open arms of the EPM) are categorized as PTSD-like, whereas rats that explore the open and closed arms of the EPM are categorized as Resilient. Thus, the Lewis rat model of PTSD provides an efficient and practical model that lends itself to a variety of experimental procedures.

### Impact of stressors on conditioned fear

As reviewed above, two key features of human PTSD are deficient extinction and impairment in hippocampal-dependent tasks. Importantly, while the latter predates trauma (Gilbertson et al., 2002, 2007), the former develops as a result of trauma (Milad et al., 2008), but only in susceptible individuals. The following two sections examine whether animal models of PTSD replicate these salient features of the human syndrome.

In numerous animal models of PTSD, exposure to a severe physical stressor leads to abnormal regulation of conditioned fear, whether the CS is a specific cue or a context. Using unsignaled footshocks, Fanselow and colleagues developed a model called *stress-enhanced fear learning*. In this paradigm, rats are pre-exposed to intense footshocks (15 shocks, 1 mA, 1 s, 4–8 min variable inter-shock interval) in context A, which results in enhanced fear responses when a single shock is later presented in context B (Rau et al., 2005; Rau and Fanselow, 2009). When contextual freezing is tested the next day in the absence of shock, or even after context A extinction training, fear levels in context B remain elevated, and this effect lasts for 3 months (Rau et al., 2005; Rau and Fanselow, 2009). These studies suggest that pre-exposure to a stressor can enhance fear sensitivity making it easier to generate new fear associations that are difficult to extinguish.

The impact of physical stressors on conditioned fear is also evaluated with the inhibitory avoidance task. In these experiments, animals are placed in a chamber that is divided in two by a wall with a door. One side is illuminated, and the other side is dark, with rats preferring the latter compartment. However, when animals are exposed to footshocks in the dark compartment, they exhibit avoidance of the dark compartment in favor of the brightly lit area. Exposure of rats to SPS impairs extinction of this inhibitory avoidance memory (Ganon-Elazar and Akirav, 2012). Paralleling these results, rats previously exposed to SPS show deficient extinction of contextual fear (Takahashi et al., 2006; Yamamoto et al., 2008), and this effect is exacerbated by neonatal isolation (Imanaka et al., 2006). However, while many studies demonstrate that footshock stress leads to enhanced contextual fear conditioning, immobilization stress does not (Daviu et al., 2010). Yet, prior immobilization/restraint stress (Chauveau et al., 2012) and SPS (Knox et al., 2012) impair extinction of conditioned responses to a cue.

Psychosocial stressors, alone or in combination with maternal deprivation, also lead to abnormal regulation of conditioned fear responses. For instance, maternal separation stress in young animals causes enhanced persistence and impaired extinction of conditioned fear (Callaghan and Richardson, 2013). Similarly, social defeat leads to enhanced fear to the conflict-paired context (Walker et al., 2008). Interestingly, enhanced acquisition and impaired extinction of contextual, but not cued, fear was also induced by social isolation in mice (Pibiri et al., 2008). In contrast, social defeat also impairs extinction of conditioned fear responses to a cue (Narayanan et al., 2011).

Of particular interest are studies addressing individual differences in response to cat odor exposure. These studies find that rats classified as “susceptible” are also impaired at the extinction of contextual fear conditioning, compared to “Resilient” rats (Nalloor et al., 2011). Surprisingly, only one study tested whether abnormalities in the regulation of conditioned fear responses predate the onset of a PTSD-like state in animals. In this study (Goswami et al., 2010), two groups of Lewis rats were subjected to auditory fear conditioning, extinction training, and testing, either before or after predator threat. Exploratory behavior on the EPM 1 week after predator threat was used to classify subjects as resilient or PTSD-like, as described above. When predator threat occurred prior to fear conditioning and extinction training, PTSD-like and Resilient rats exhibited similar levels of freezing to the auditory CS by the end of training and at the onset of the recall test the next day. However, with additional presentations of the CS, PTSD-like rats showed impairments in within-session and between-session extinction. In contrast, when predator threat followed fear conditioning, resilient, and PTSD-like rats displayed indistinguishable levels of fear expression during all phases of the fear conditioning protocol. This indicates that the fear extinction deficit of Lewis rats is acquired after exposure to predator threat, reproducing the extinction impairments observed in the human syndrome.

### Abnormalities of hippocampal structure and function

Many animal models of PTSD are associated with signs of abnormal hippocampal structure and function, as seen in the human syndrome. While this supports the face validity of these models, these similarities may be coincidental and depend on different mechanisms. We return to this question below. A large body of research shows that various models of chronic stress lead to neuronal atrophy within the hippocampus, and decreased cell proliferation in the dentate gyrus (Watanabe et al., 1992; Magarinos and McEwen, 1995; Conrad et al., 1996; Magarinos et al., 1996; Vyas et al., 2002; Kikuchi et al., 2008; Tamaki et al., 2008; Li et al., 2010). In contrast, chronic stress leads to increased dendritic branching and in dendritic spine numbers within the amygdala (Vyas et al., 2003; Mitra et al., 2005). However, the precise mechanisms by which hippocampal abnormalities contribute to impaired performance on memory and cognitive tasks remains unclear. As Samuelson (2011) suggests, these memory deficits may be explained by deficient processes in the pre-frontal cortex. In contrast, Zoladz and Diamond (2013) emphasize the complex, and at times, contradictory pattern of relationships between hippocampal dysfunction and cognitive tasks, and suggest the role of the hippocampus eludes simple characterization. These concerns notwithstanding, if animal models of PTSD reproduce findings in humans regarding the hippocampal-dependent tasks, this lends support to the face validity of these models.

Consistent with the evidence of hippocampal atrophy, many animal models of PTSD are also associated with signs of hippocampal dysfunction. For instance, immobilization stress (Andero et al., 2012) and underwater trauma (Richter-Levin, 1998; Wang et al., 2000) impair spatial memory on the Morris water maze. Similarly, 1 week after exposure to SPS, rats are impaired on a novel object recognition (NOR) task (Wang et al., 2012).

The available data regarding the impact of psychosocial stress on hippocampal-dependent behavior is consistent with that of physical stressors. For instance, chronic social instability combined with predator stress exposure impairs spatial memory (Diamond et al., 1999; Park et al., 2008), and NOR (Zoladz et al., 2008). Also, acute exposure to predator stress alone selectively impairs hippocampal-dependent working memory, while sparing hippocampal-independent reference memory in the radial arm water maze (Woodson et al., 2003).

Much evidence suggests that the hippocampal abnormalities seen in chronic stress models of PTSD are due to the neurotoxic effects of corticosteroids. However, it is unclear whether the same mechanisms are operative in PTSD. First, results regarding baseline and stress levels of circulating corticosteroids in PTSD are contradictory (Meewisse et al., 2007; Klaassens et al., 2012). The only consistent finding is that compared to controls, PTSD subjects display an enhanced suppression of cortisol release in response to dexamethasone (Yehuda et al., 1993; Griffin et al., 2005; Golier et al., 2006). As a result, it may be incorrect to assume that high circulating levels of corticosteroids are responsible for the hippocampal abnormalities in PTSD. Second, it now appears that the hippocampal atrophy seen in PTSD predates trauma since it is observed in non-traumatized monozygotic twins (Gilbertson et al., 2002). Thus, while the hippocampal alterations observed in animal models appear similar to PTSD, they may depend on different mechanisms.

Confidence that the hippocampal alterations seen in animal models of PTSD and in the human disorder depend on similar mechanisms would be increased if they were apparent prior to the traumatic event, as in human PTSD. Although it remains unclear whether this is the case for hippocampal volumes (Golub et al., 2011), there is evidence that hippocampal function is altered in susceptible rodents prior to the stressor. For instance, mice with high pre-stressor levels of N-acetylaspartate (NAA) in the left hippocampus showed decreased contextual fear and arousal compared to animals with lower levels of NAA (Siegmund et al., 2009). Moreover, evidence of impaired hippocampal function before trauma was obtained in susceptible Lewis rats (Goswami et al., 2012).

In rodents, hippocampal function is often assessed using variations of recognition memory paradigms. In previous studies, rats with ibotenic acid lesions of the entire hippocampus could identify novel objects normally. However, they are impaired in their ability to recognize novel spatial configurations of these objects in an allocentric frame of reference (Langston and Wood, 2010). Building on these findings, a recent study compared the performance of resilient vs. PTSD-like Lewis rats on three types of recognition memory tasks that vary in their hippocampal-dependence (Goswami et al., 2012). In these tasks, preferential exploration of novel relative to familiar items is used to assess recognition memory. In one task, the NOR task, the rats could simply use object identity to detect item novelty. In the other two tasks, object identity and locations had to be combined to determine item novelty in either an egocentric (egocentric object recognition task—EOR) or an allocentric (allocentric object recognition task—AOR) frame of reference. As mentioned above, only the latter task (AOR) is thought to be dependent on the hippocampus (Langston and Wood, 2010). Three separate groups of Lewis rats were subjected to one of the three tasks, prior to predator threat. Exploratory behavior on the EPM was then used to classify rats in resilient vs. PTSD-like groups. The performance of Resilient and PTSD-like rats was indistinguishable on the NOR and EOR tasks. In contrast, PTSD-like rats were impaired on the AOR task.

These results suggest that even prior to trauma, PTSD-like rats show a deficit in hippocampal-dependent functions, paralleling human findings. However, whether hippocampal structure is also altered in susceptible rats remains to be determined. Nevertheless, combined with the fear extinction deficit described above (Goswami et al., 2010), and the individual variations in trauma susceptibility, the evidence of impaired hippocampal function suggests that the Lewis rat model of PTSD has face and ecological validity.

## Conclusions

PTSD is a complex disorder that affects a proportion of individuals who experience a traumatic event. Given the variety of events that can trigger PTSD, and the wide range of symptoms that arise, it is unlikely that a single animal model will reproduce the complexity of the human disorder. Nevertheless, the evidence reviewed above suggests that some animal models do mimic core aspects of human PTSD including individual differences in susceptibility to various stressors, avoidance, hyperarousal, fear dysregulation (generalization and deficient extinction) as well as hippocampal dysfunction. While many models reproduce some aspects of human PTSD, in our opinion, the predatory threat model comes closest to the human syndrome. Indeed, in addition to reproducing avoidance, anxiety, and individual differences in trauma susceptibility, this model mimics the fear extinction and hippocampal processing deficits seen in the human syndrome, *including their different temporal relationship to trauma*. A challenge for future studies will be to combine this model with analytical techniques such as single-unit recordings in behaving animals or *ex vivo* patch-clamp investigations of neuronal properties in PTSD-like and resilient subjects, before vs. after trauma. This will further our understanding of the neuronal mechanisms responsible for the induction and maintenance of PTSD. As a result, our understanding of the etiology of PTSD may be enhanced, paving the way for improved preventive and therapeutic interventions.

### Conflict of interest statement

The authors declare that the research was conducted in the absence of any commercial or financial relationships that could be construed as a potential conflict of interest.

## Key Concept

Post-traumatic stress disorder: a debilitating anxiety disorder that develops in a proportion of individuals following a traumatic event that produced feelings of intense fear, horror, or helplessness and is characterized by re-experiencing of the trauma, avoidance, and hyperarousal.
Classical fear conditioning: The leading laboratory model for studying how organisms learn to predict danger by experience. In this model, a neutral conditioned stimulus (CS), such as a context or tone, is paired with a noxious unconditioned stimulus (US), typically a mild electrical shock to the hand or wrist. After a few CS-US pairings, presentation of the CS alone comes to elicit conditioned fear responses.
Allocentric: A frame of reference where individuals must use the spatial relationships between external cues in order to successfully navigate within their environment.
Egocentric: A frame of reference where the location of objects is defined with respect to the observer's body axes (left-right; up-down; front-back).
Fear extinction: Repeatedly presenting the CS in the absence of US reduces conditioned fear. Extinction does not erase the fear memory, but leads to the formation of a new inhibitory memory. After extinction, fear responses return with time or when the CS is presented in a context different than where extinction training occurred. These properties of extinction account for the limited efficacy of exposure therapy.
Physical stressors: A stressor involving physical harm or discomfort.
Psychosocial stressors: In some PTSD models, animals are subjected to a variety of psychosocial stressors such as housing instability, social defeat, social isolation, and separation from the social group. These models attempt to reproduce an important risk factor for human PTSD.
Early life stressors: In humans, stress exposure early in life pre-disposes individuals to be more susceptible to subsequent stressors. Some animal models attempt to reproduce this using maternal separation or other stressors early during development. As in humans, juvenile animals subjected to early life stressors are more likely to develop extreme anxiety-like phenotypes when exposed to stress again in adulthood.
Psychogenic stressors: An event perceived as a severe threat in the absence of physical harm or discomfort.
Predator threat or stress: In some PTSD models, rodents are exposed to species-relevant predators (predator stress) or their odor (predator threat). A major strength of these models is their ecological validity.
